# Proximal and distal predictors of self-regulatory change in children aged 4 to 7 years

**DOI:** 10.1186/s12887-020-02133-6

**Published:** 2020-05-18

**Authors:** Kate E. Williams, Steven J. Howard

**Affiliations:** 1 School of Early Childhood & Inclusive Education, Faculty of Education, Queensland University of Technology, QUT, Level 4 E Block, Victoria Park Road, Kelvin Grove, QLD 4059 Australia; 2 Early Start, School of Education, Faculty of Social Sciences, University of Wollongong, Wollongong, Australia

**Keywords:** Early childhood, Self-regulation, Self-control, Predictive model

## Abstract

**Background:**

Growth in early self-regulation skills has been linked to positive health, wellbeing, and achievement trajectories across the lifespan. While individual studies have documented specific influences on self-regulation competencies in early childhood, few have modelled a comprehensive range of predictors of self-regulation *change* across health, development, and environment simultaneously. This study aimed to examine the concurrent associations among a range of proximal and distal influences on change in children’s self-regulation skills over 2 years from age 4–5 years.

**Methods:**

Data from the Longitudinal Study of Australian Children (*N* = 4983) were used in a structural equation model, predicting a multi-source composite measure of self-regulation at each of 4–5 years and 6–7 years. By controlling for earlier self-regulation and covariates, the model examined the relative contributions of a comprehensive range of variables to self-regulation change including health, development, educational, home environment, time-use, and neighbourhood characteristics.

**Results:**

The significant predictors of children’s self-regulation growth across 4 to 7 years were fewer behavioural sleep problems, higher gross motor and pre-academic skills, lower levels of maternal and paternal angry parenting, and lower levels of financial hardship. There were also marginal effects for high-quality home learning environments and child-educator relationships.

**Conclusion:**

Findings suggest that if we are to successfully foster children’s self-regulation skills, interventionists would do well to operate not only on children’s current capacities but also key aspects of their surrounding context.

## Background

Self-regulation refers to the ability to exert control over our cognition, emotion, and behaviour in ways that are adaptive to functioning. These skills develop across the lifespan, but most rapidly in early childhood alongside cortical maturation processes. In terms of self-regulation development, early improvements appear to be better, with strong early childhood self-regulation skills linked with a wide range of health and achievement outcomes across the lifespan, including positive mental and physical health, and educational attainment [1–3]. In contrast, poorer self-regulation in early childhood has been linked with school adjustment difficulties [4], behaviour problems [5], adolescent risk-taking [2], and adult disordered behaviour [6].

Early childhood is a period in which growth in self-regulation is not only particularly desirable, but also demonstrably possible. In fact, *growth* in self-regulation skills in the early years of life (controlling for early self-regulatory levels and family environment) has been found to reduce risk of childhood behaviour problems [5], adolescent crime, self-harm, and mental health problems [2], as well as enhance academic learning trajectories [7]. Given limited understanding of antecedents of early self-regulation change that can shift trajectories and outcomes more broadly, intervention approaches remain incongruous. For instance, approaches to self-regulation intervention include computerized executive function training, specialized preschool curricula, physical activities, arts and music, motor skill development, and so forth [8–10]. While it is indeed likely that multiple approaches will be effective, and ideally suited to different contexts, needs and children, the design of interventions would nevertheless be improved through a more comprehensive and holistic understanding of early childhood factors and experiences that support self-regulation development.

The individual and environmental conditions that support optimal development in self-regulation across early childhood remain relatively unclear. Various lines of inquiry have identified longitudinal predictors associated with better point-in-time self-regulation in early childhood including rich home learning environments [11], positive parenting approaches [12], stronger motor [13] and language development [14], and well-adjusted sleep behaviours [15]. However, very few studies have examined the extent to which these, and other plausible proximal and distal factors predict *change* in self-regulation over time. The aim of this study is to investigate the concurrent associations among a range of proximal and distal influences on change in children’s self-regulation skills over 2 years beginning at 4–5 years of age.

## Methods

### Participants

This study used data from the population-representative Kindergarten (K) cohort of the *Longitudinal Study of Australian Children* (LSAC), with full study design details described elsewhere [16]. In brief, for the K cohort, 4983 children aged 4–5-years old were recruited in 2004 with biennial data collection occurring since then. Data collection involves parent and teacher questionnaires, computer assisted interviews with parents and children, and direct assessments with children. The current study uses data collected for the K cohort across two waves (when children were 4- to 5-years old and 6- to 7-years old). Table 1 describes the characteristics of the sample.

**Table 1:** Sample characteristics

Study sample characteristic	Percentage
Boys	51%
English as main home language	86%
Aboriginal or Torres Strait Islander	3.8%
Mothers with incomplete high school education	22%
Mothers with university education	28%
Attending preschool program at 4–5 years	95%
	**M (SD)**
Child age at 4–5 year data collection	56.9 months (2.65)
Child age at 6–7 year data collection	81.9 months (2.96)
Household income per week	$1661.93AUD ($1294.05)

### Measures

*Self-regulation* was assessed at 4–5 and 6–7 years of age using a factor score we have previously established as a reliable indicator of children’s self-regulatory capacity with good predictive validity of broad later-life outcomes into adolescence [2]. A total of 20 survey items from parent-, teacher-, and observer-report ratings of self-regulation were standardized and then averaged to create a single composite score (*M* = 0, *SD* = 1). Constituent items of this factor index the extent to which children can control and sustain their attention, and control their behaviour and emotions (see Table 2). Internal consistency was high (alpha = 0.84 at 4–5 years, 0.86 at 6–7 years).

**Table 2:** Items included in the self-regulation measure at 4–5 years and 6–7 years

Construct	Respondent	Item
Impulsive Aggression	Parent and teacher	Often has temper tantrums/hot tempers
	Parent and teacher	Often fights with other children or bullies them
	Parent and teacher	Often argumentative with adults
Hyperactivity	Parent and teacher	Restless, overactive, cannot stay still for long
	Parent and teacher	Constantly fidgeting or squirming
	Parent and teacher	If this child is upset, it is hard to comfort him/her
Lack of Persistence & Inattention	Parent and teacher	The child likes to complete one task or activity before going on to the next (reversed)
	Parent and teacher	Sees takes through to the end, good attention span (reversed)
	Parent and teacher	The child stays with an activity (e.g., puzzle, construction, kit, reading) for a long time (reversed)
	Parent, teacher, and observer	Easily distracted, concentration wanders
Impulsivity	Parent and teacher	Can stop and think things out before acting (reversed)
	Parent and teacher	Shares readily with other children (reversed)
	Observer	Degree of negative mood (withdrawn, uncooperative, sulky, seeming upset, angry) to interview

*Predictors of self-regulation change* were selected from the domains of health, development, home environment, education, time use, and neighbourhood measured when children were 4–5-years old. Details of each of these are provided in Table 3. Where parent-report is indicated, this was provided by Parent 1 (defined by LSAC as the parent who knows the study child best, which in 97% of cases was the mother).

**Table 3:** Predictors of self-regulation growth in the model

Construct	Data source	Measure
**Health & Health Behaviours**		
Physical health	Parent	Physical Health Summary score from the *Pediatric Quality of Life Inventory* (PedsQL) [17]. Summed and average score of 8 items each rated on 5-point scale, tapping a child’s level of functioning in daily activities that rely on good physical health. E.g. *problems with running. α* = .72
Diet quality	Parent	Units of high sugar drinks consumed in the last week
Behavioural sleep problems	Parent	Five items modelled as a latent variable as per prior studies [18]. E.g. child has problems on 4 or more nights a week with waking during the night (yes/no); this child’s sleep is a small/moderate/large problem.
**Development**		
Receptive vocabulary	Assessed	*Peabody Picture Vocabulary Test* [19] of receptive vocabulary in which children listen to a spoken word and are asked to point to the matching picture given a set of four pictures. Higher scores represent higher receptive vocabulary skills.
Gross motor development	Teacher	On a 4-point scale from ‘much less competent than peers’ to ‘more competent than peers’
Fine motor development	Teacher	On a 4-point scale from ‘much less competent than peers’ to ‘more competent than peers’
Pre-academic skills	Assessed	*Who Am I* test [20]. Children write their names, copy shapes, write words and numbers; scored according to skill level. *α* = .89 [21]
Home environment		
Maternal parenting anger	Mother	Composite measure (weighted mean score) as per LSAC technical advice [22] using four adapted items from the National Longitudinal Study of Children & Youth [23]. Each item rated on 5-point scale from ‘never or almost never’ to ‘almost always’. E.g. how often are you angry when you punish this child? *H* = .72.
Paternal parenting anger	Father	
Maternal parenting consistency	Mother	Composite measure as per LSAC technical advice [22] using five items from the National Longitudinal Survey of Children and Youth [23]. Each item rates on a 10-point scale from ‘not at all’ to ‘all of the time’. E.g. how often does this child get away with things that you feel should have been punished? *H* = .80 for father; .82 for mothers.
Paternal parenting consistency	Father	
Maternal mental health	Mother	*Kessler K6* screening scale [24] of six items (summed and averaged) about respondents’ feelings over the past four-week period. Rates on 5-point scale from ‘all of the time’ to ‘none of the time’. E.g. in the past 4 weeks how often have you felt hopeless? *α =* .84 for mothers, .82 for fathers.
Paternal mental health	Father	
Home learning environment	Parent	Single item book reading; plus latent variable with five indicators of other home learning activities including music, art, and play as used in other LSAC studies [25]. Each rated on 4-point scale of frequency of adult-child engagement for each activity in the last week from ‘not in the past week’ to ‘6–7 days in the week’.
Financial hardship	Parent	7-item count index ranging from 0 to 7, based on summing Yes = 1, No = 0 responses to 7 items including couldn’t pay bills, gone without meals as used in prior LSAC research [26].
Argumentative parental relationships	Parent	Composite of 5 items (summed and averaged) rated on a 5-point scale from ‘never’ to ‘always’. E.g. my partner and I argue; disagree over child-rearing etc. *α =* .80
Stressful life events	Parent	13-item count index ranging from 0 to 13 based on summing Yes = 1, No = 0 responses about exposure to adverse life events over the past year including marital breakdown, death of friend, as per prior LSAC research [27].
**Education**		
Teacher-child relationship	Teacher	8-item composite drawn from the *Student Teacher Relationship Scale* [28] following prior LSAC factor modelling [29]. Each item rated on 5-point scale from ‘definitely does not apply’ to ‘definitely applies’. E.g. share affectionate relationships, easy to be in tune with feelings *α =* .81
**Time use**		
Extra-curricular sport	Parent	Sum of 3 items indicating participation (yes / no) in extra-curricular swimming, gymnastics, or team sport
Extra-curricular music / dance	Parent	Sum of 2 items indicating participation (yes / no) in extra-curricular music and dance
Weekday TV hours	Parent	Number of hours watching TV on a typical weekday
Weekday computer hours	Parent	Number of hours using a computer on a typical weekday
Physical activity	Parent	Parent-rated child enjoyment of physical activity on a 5-point scale from ‘very much dislikes physical activities’ to ‘very much likes physical activity’
**Neighbourhood**		
Liveability	Parent	Composite (sum) of 8 items each rated on 4-point scale from ‘strongly disagree’ to ‘strongly agree’. E.g. this is a safe neighbourhood, this neighbourhood has good parks. *α =* .76
Socio-economic index for area (SEIFA)	Australian Bureau of Statistics	Composite of 31 variables (e.g. income, unemployment, occupation and education) computed by the Australian Bureau of Statistics [30].

*Control variables* included in the analyses were gender, age of assessment (in months) at baseline, birth weight percentile, whether or not the child had ever been breastfed, Aboriginal and Torres Strait Islander status, Non-English speaking home background, maternal education level (on a 6-point scale from incomplete high school to postgraduate degree), and household income bracket. We used data from the age 4–5 years data collection for these variables, providing the most complete data possible (before attrition in the longitudinal study).

### Approach to analysis and missing data

**Fig. 1:**
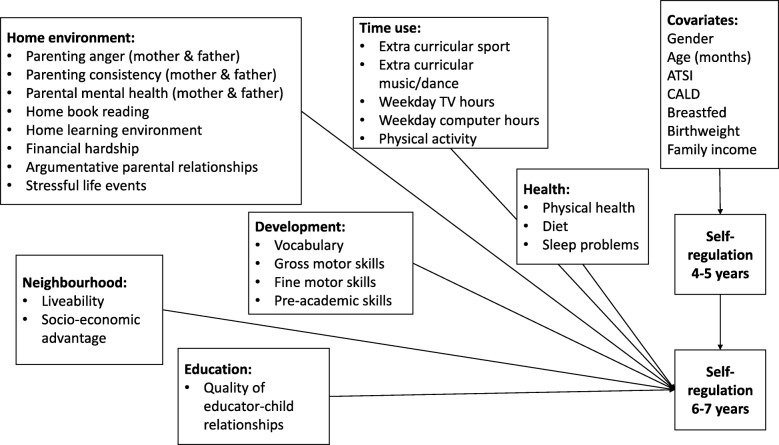
Conceptual model tested through structural equation model analyses

A structural equation model was tested in Mplus version 7.11. Figure 1 depicts the model, showing self-regulation at 6–7 years predicted by the full range of variables described above, while controlling for self-regulation measured two years earlier. This approach to modelling means the estimates for the predictors represent their impact on residualized change in self-regulation from 4 to 7 years of age, because the effect of the earlier measure of self-regulation has already been accounted for. Additionally, effects of stable covariates present from birth on earlier self-regulation were controlled for. Correlations among all predictor variables were included in the model, with the strongest significant correlation as r = .45 for the correlation among teacher-reported gross motor and fine motor skills. Due to the large sample size, we use a conservative *p* value of < .01 to indicate a significant effect and < .02 for a marginally significant effect.

The amount of missing data varied across waves and variables, ranging from no missing data for socio-demographic characteristics at 4–5 years to 45% missing data for the self-regulation scores at 4–5 and 6–7 years due primarily to item-level missing data from teacher non-report. The data were considered missing at random (MAR) because it was unlikely that the presence of a missing value was related to the response that would have been given [31]. We used full information maximum likelihood with a robust estimator to address missing data, allowing us to retain 98% of the sample in the statistical models. We used the sampling weights provided for LSAC [32] to account for sampling error.

## Results

The model was a good fit for the data and accounted for 42% of variance in self-regulation at 6–7 years, with all estimates shown in Table 4. Self-regulation skills at 6–7 years, after controlling for self-regulation skills at 4–5 years, were predicted by fewer behavioural sleep problems, higher gross motor and pre-academic skills, lower levels of maternal and paternal angry parenting, and lower levels of financial hardship. There were also marginal effects for the home learning environment and child-educator relationships. Covariates associated with stronger self-regulatory skills at 6–7 years including being a female, having a higher birthweight percentile, identifying as non-Aboriginal and Torres Strait Islander, having a mother with a higher level of education, and a higher household income.

**Table 4:** Standardized coefficients for the predictors of self-regulation at 6–7 years controlling for prior self-regulation and covariates

	*β*	95% CI
***Covariate associations with self-regulation at 4–5 years***		
Female	.50**	.44–.57
Age	.01	.04–.11
Birthweight percentile	.07**	.03–.11
Breastfed	−.15	−.29 - -.01
Aboriginal Torres Strait Islander	−.53**	−.81 - -.26
Non-English home language	.01	−.10–.11
Maternal education level	.13**	.09–.17
Household income	.13**	.08–.17
***Stability of self-regulation 4–5 years to 6–7 years***	.54**	.49–.59
***Predictors of self-regulation at 6–7 years controlling for above***		
*Health*		
Physical health status	.02	−.03–.05
High sugar drink intake	.02	−.01–.06
Sleep problems	−.08**	−.13 - -.04
*Development*		
Vocabulary	.01	−.03–.06
Gross motor	.06**	.02–.10
Fine motor	−.05	−.10–.00
Pre-academic skills	.12**	.09–.16
*Home environment*		
Maternal angry parenting	−.10**	−.15–.06
Paternal angry parenting	−.12**	−.16 - -.07
Maternal consistent parenting	−.01	−.04–.05
Paternal consistent parenting	.02	−.02–.07
Maternal mental health	−.01	−.06–.04
Paternal mental health	.02	−.03–.06
Shared book reading frequency	.03	−.01–.07
Home learning activities	.06*	.01–.10
Financial hardship	−.07**	−.12 - -.02
Argumentative parental relationships	−.03	−.07–.02
Stressful life events	−.00	−.04–.04
*Education*		
Educator-child relationship	.06*	.01–.11
*Time use*		
Extra-curricular sport	−.02	−.05–.02
Extra-curricular music/dance	.02	−.01–.05
Weekday TV hours	.04	.00–.08
Weekday computer hours	.01	−.03–.05
Physical activity	−.03	−.06–.00
*Neighbourhood*		
Liveability	−.01	−.05–.02
Socio-economic index	.01	−.03–.04

* *p* < .02; ** *p* < .01

## Discussion

This is the first paper to model a comprehensive and concurrent set of predictors across health, development, and environment in relation to self-regulatory development of young children, across a two-year period beginning from age 4–5 years. Controlling for a range of background factors, significant predictors of self-regulatory growth included: fewer behavioural sleep problems; higher gross motor and pre-academic skills; lower levels of maternal and paternal angry parenting; lower levels of financial hardship; and marginal effects for home learning environment and child-educator relationships. As predictors were modelled simultaneously, significant findings provide a (likely conservative) estimate of the associations between each variable and self-regulation change, over and above the combined associations of all other variables in the model. While previous studies have provided insight into the transactional mechanisms between some factors known to influence self-regulation (e.g. parenting and sleep), this model better reflects the complexity of children’s lives and the combined impact of a range of factors on self-regulatory *change*. Thus the study makes an important contribution toward prevention and intervention efforts by identifying the most salient and high-potential factors to target for self-regulation interventionists taking a holistic approach to supporting self-regulatory growth in young children.

Substantial research and theory supports both acute and persistent associations of self-regulation with learning and academic skills [33] with self-regulation typically positioned as a *predictor* of academic skills. In a related finding, but with self-regulation as the *outcome*, in our model pre-academic skills were one of the strongest predictors of self-regulation growth. It is clear why self-regulation would predict learning and academic skills: the ability to direct and sustain attention, tackle new challenges, resist maladaptive impulses, and work collaboratively and pro-socially with others – all hallmarks of high self-regulation – serve to support on-task behaviour, effort and persistence during learning. However, there is comparatively less research focused on the possible reciprocal effects with pre-academic skills predicting self-regulation growth. A number of explanations are feasible. First, it is likely that self-regulation and early literacy and numeracy skills, as represented by our pre-academic skill assessment, develop in a bidirectional manner across early childhood [34, 35]. For example, time spent in focussed literacy and numeracy learning activities provides the opportunity to extend and enhance self-regulatory capacities, particularly in attentional and cognitive control aspects. It is likely that had we had an earlier and multiple measures of both self-regulation and early concept comprehension, literacy, and numeracy, we would have established birdirectional and reciprocal associations across time. A second and related explanation is that the pre-academic assessment used here may have tapped children’s visual-motor skills given it was a pencil and paper task requiring the writing of letters. While there was no visual-motor data available for children in this dataset, scores on the pre-academic test did correlate (*r* = .40) with the fine motor variable in our model (single item of teacher report of fine motor competence). Recent research has suggested that visual-motor skills and cognitive self-regulation, as enabled by executive functions, co-develop in a bidirectional manner [35] and it may be that our findings are reflecting a small portion of this transactional process at this period of development. That is, children who scored more highly on the pre-academic score may have done so due to higher visual-motor skills, which may themselves co-develop with and support self-regulatory growth.

Pre-school gross motor abilities were also significantly, albeit modestly, associated with children’s self-regulation growth. This is consistent with suggestions of common mechanisms (i.e., executive functions) that are implicated in both self-regulation and motor learning [36–38], such that both show common areas of neural activation, are impaired after damage to neural regions for the other, and are often both impaired in cognitive disorders, such as ADHD and dyslexia. Indeed, tasks that are motor-demanding for young children, such as navigating uneven surfaces and/or obstacles, are more cognitively demanding and lead to more cognitive errors than less cognitively demanding motor tasks [39]. As such, one possibility is that this finding is indicative of the concomitance between self-regulatory and motor skills. However, that gross motor skills were associated with *change* in self-regulation may additionally suggest that the acquisition of motor proficiency creates new learning opportunities [40] such as experiences that serve to foster self-regulation (e.g., increased mobility causing children to encounter rules associated with access, involvement in physically active shared play providing opportunities for impulse control and turn-taking, etc.) As such, gross motor skills may open a gateway to important self-regulation-promoting experiences and activities, whereas low levels of gross motor skills might consume much of the cognitive resource that otherwise could be directed toward these same activities.

Another factor that was modestly but significantly and uniquely related to self-regulation growth was sleep problems. This aligns with a large body of existing research that identifies sleep problems as a key contributor to daytime self-regulatory problems in young children both in the short [41] and long term [18, 42]. It is possible that behavioural sleep problems in young children reflect an underlying phenotype associated with regulatory problems [43, 44], and/or that early behavioural sleep problems initiate a developmental cascade that disrupts emotional and attentional development over time [15]. Either way, brief sleep interventions are known to be safe and effective in improving both sleep behaviours and daytime self-regulatory functioning in young children in both typically-developing [45–47] and clinical populations [48, 49].

Our finding that angry parenting was associated with less growth in self-regulation for children echoes a range of prior studies that have linked aggressive, controlling parenting with poor self-regulation in children [50–54]. However, this study extends that work by including not only mothers’ but also fathers’ parenting, a rare inclusion. We suggest that angry parenting as measured here is indicative of dysregulated parenting, and potentially of overall emotional regulation skills of parents. Mechanisms through which this might limit self-regulatory growth in children include heritability pathways in terms of self-regulation capabilities [55], and socialisation pathways in which children learn about self-regulatory behaviours through modelling their parents’ behaviours. It is also important to note that child-driven effects are possible, as reflected in prior studies that show dysregulation in young children is associated with increased parenting stress and more-negative parenting approaches [56, 57]. These bidirectional relationships between parenting and children’s self-regulation, which are likely to establish mutual promotion/exacerbation processes over time, were not modelled in this study and should be the focus of future longitudinal work.

A number of socioeconomic variables were associated with enhanced self-regulatory growth including higher household incomes, higher maternal education levels and living in households with lower levels of financial hardship. The experience of significant financial hardships such as those tapped here is likely associated with stressful home environments, which impact on children’s physiology and neurodevelopment in ways that limit their capacity for self-regulation development [58, 59]. Indeed, early self-regulation has been identified as one of the foremost mechanisms through which early stressors and socioeconomic disadvantage can lead to poorer academic and wellbeing outcomes [60]. For these reasons, much of the prevention and intervention focus to date has been on children from disadvantaged backgrounds in an effort to address socio-economic gradients in achievement likely mediated through early self-regulatory capacity. Our findings suggest this focus is well-placed.

Marginal effects were also found for the association between educator-child relationships and the home learning environment, with self-regulatory change. The finding regarding importance of educator-child relationship in terms of children’s early self-regulation development reflects other similar findings in both Australia [61] and Europe [62]. Positive student-teacher relationships likely matter because they set the context within which teachers can enact strategies particularly important for acquiring self-regulation during the preschool developmental period [63] including co-regulation, modelling and coaching [64]. Our findings regarding the home learning environment align with a prior American longitudinal study linking parental involvement in home learning activities with children’s self-regulatory development [65].

### Limitations

Although this study included a comprehensive array of predictors of self-regulation growth across a specific period in early childhood, there are a number of limitations related primarily to measurement. Most measures were broad and blunt instruments of their constructs. This reflects the nature of the population dataset, in which a broad spectrum of measures capturing child development and the environment were desired, rather than an in-depth measurement of any particular constructs. In addition, our self-regulation composite was only available at two time points in this dataset, meaning that more sophisticated growth curve modelling, which requires a minimum of three time points, could not be undertaken. It is also important to note that although we included a wide array of predictors, nearly 60% of the variance in our self-regulation composite at 6–7 years was still unexplained by the model. This suggests that even large-scale studies such as these are missing key ingredients related to self-regulatory growth. Our understandings could be enhanced through studies which capture potential variables that are not often measured, including chronic stress (e.g. cortisol), psychophysiological arousal and regulation, sensory processing, and more detailed understandings of the nature of home learning and early education and care activities. Finally, it is important to note that participants in this study were recruited in 2004. While it is anticipated that there has been limited change in most lifestyle factors investigated (e.g., parenting), new cohort studies are required to better understand the influence of more prominent societal change such as increased access and use of digital devices.

## Conclusion

While we know that self-regulation is important for a broad range of longitudinal achievement and wellbeing outcomes, and that early childhood is a key window for self-regulatory growth, we have not yet been overly effective in intervention efforts. One reason for this might be that we need more holistic and evidence-informed theories and approaches to self-regulatory development, rather than a focus on single factors that appear predictive in isolation. We need more complex modelling of the interactions between these various factors and their association with self-regulation change (not just prediction at one time point). The findings of this study suggest a starting point for further detailed research that aims to achieve this.

## Data Availability

The dataset analysed for the current study is available from the Australian Data Archive https://dataverse.ada.edu.au/dataset.xhtml?persistentId=doi:10.26193/JOZW2U

## Abbreviations

K cohort: Kindergarten cohort
LSAC: Longitudinal Study of Australian Children
